# Seasonal Water Quality and Algal Responses to Monsoon-Mediated Nutrient Enrichment, Flow Regime, Drought, and Flood in a Drinking Water Reservoir

**DOI:** 10.3390/ijerph182010714

**Published:** 2021-10-13

**Authors:** Md Mamun, Usman Atique, Ji Yoon Kim, Kwang-Guk An

**Affiliations:** Department of Bioscience and Biotechnology, Chungnam National University, Daejeon 34134, Korea; mamun1006001@gmail.com (M.M.); physioatique@gmail.com (U.A.); jiyoonn20@naver.com (J.Y.K.)

**Keywords:** seasonal trends, multivariate analysis, drought, mesotrophic, drinking water, reservoir

## Abstract

Freshwater reservoirs are a crucial source of urban drinking water worldwide; thus, long-term evaluations of critical water quality determinants are essential. We conducted this study in a large drinking water reservoir for 11 years (2010–2020). The variabilities of ambient nutrients and total suspended solids (TSS) throughout the seasonal monsoon-mediated flow regime influenced algal chlorophyll (Chl-a) levels. The study determined the role of the monsoon-mediated flow regime on reservoir water chemistry. The reservoir conditions were mesotrophic to eutrophic based on nitrogen (N) and phosphorus (P) concentrations. An occasional total coliform bacteria (TCB) count of 16,000 MPN per 100 mL was recorded in the reservoir, presenting a significant risk of waterborne diseases among children. A Mann–Kendall test identified a consistent increase in water temperature, conductivity, and chemical oxygen demand (COD) over the study period, limiting a sustainable water supply. The drought and flood regime mediated by the monsoon resulted in large heterogeneities in Chl-a, TCB, TSS, and nutrients (N, P), indicating its role as a key regulator of the ecological functioning of the reservoir. The ambient N:P ratio is a reliable predictor of sestonic Chl-a productivity, and the reservoir was P-limited. Total phosphorus (TP) had a strong negative correlation (R^2^ = 0.59, *p* < 0.05) with the outflow from the dam, while both the TSS (R^2^ = 0.50) and Chl-a (R^2^ = 0.32, *p* < 0.05) had a strong positive correlation with the outflow. A seasonal trophic state index revealed oligo-mesotrophic conditions, indicating a limited risk of eutrophication and a positive outcome for long-term management. In conclusion, the Asian monsoon largely controlled the flood and drought conditions and manipulated the flow regime. Exceedingly intensive crop farming in the basin may lead to oligotrophic nutrient enrichment. Although the reservoir water quality was good, we strongly recommend stringent action to alleviate sewage, nutrient, and pollutant inflows to the reservoir.

## 1. Introduction

It is broadly recognized that lentic ecosystems are increasingly impacted by a plethora of pressures resulting from ongoing anthropic activity [1,2]. With the ever-increasing global population, inland water resources are increasingly threatened by challenges such as climate change [3], agricultural intensification [4], urbanization [5], pollutants [6,7], and flow-regime disturbances [8]. The drivers of changes in freshwater ecosystems are labelled as stressors. Usually, bioindicator sentinel species are used to detect the various stressors in the aquatic ecosystems [9,10]. They include altered flow regimes [11], nutrient enrichment [12], sediment loads [13], and rapidly deteriorating water quality [14]. These stressors actively influence the biodiversity of freshwater ecosystems [15,16,17,18]. Consequently, there is an urgent need to ensure that regulators, managers, and stakeholders understand the diverse assemblage of these diverse threats to water quality. These challenges can individually threaten lentic ecosystems or combine to act as multistressors; hence, their impact on drinking water facilities must be thoroughly investigated.

Due to the complex assemblage of multistressors, managing sustainable water quality in lakes and drinking water reservoirs has become a formidable challenge [19]. Therefore, it is essential to use scientific evidence to manage nutrient enrichment in large reservoirs and lakes effectively. The most common approach used to mitigate eutrophication is to control the external and internal loading of significant nutrients (mainly phosphorus (P)) to prevent excessive phytoplankton biomass production [20,21]. This approach is based on the fact that phytoplankton growth is generally P-limited in freshwater reservoirs and lakes. It is primarily established through empirical relationships determined by long-term monitoring [22,23,24]. However, the site-specific uncertainty linked with sestonic chlorophyll-a (Chl-a) levels and total phosphorus (TP) regression modelling in large lakes is widely used to predict the response of phytoplankton biomass associated with reduced TP loads [25,26,27].

Artificial reservoirs differ from natural lakes in numerous aspects, such as a shorter water residence time (WRT), recurrent water level fluctuations, and regular water abstraction [20,28,29]. This is the case with most of the nearly 18,000 human-made reservoirs in South Korea. The stressors they experience are linked to their geographic position, with several located adjacent to large cities to provide drinking water and flood mitigation [25,30]. Overall, large reservoirs face severe water quality degradation and nutrient enrichment coupled with industrial pollution, cumulatively jeopardizing human health and the provision of their intended ecological services [2,31,32].

Reservoir water chemistry fluctuations are assessed in terms of various parameters for which standards were established to indicate the range of suitability to humans and ecological systems [1]. Deviations in essential parameters, including TP, Chl-a, total nitrogen (TN), ambient nutrient ratios (N:P), total suspended solids (TSS), and chemical and biological oxygen demands (BOD, COD), are critical when outside the optimum ranges [30]. According to da Rocha Junior et al. [33], other water quality challenges are directly linked to WRT and water volume reduction, leading to an increased risk of nutrient enrichment and concomitant water quality degradation. Brasil et al. [34] highlighted the critical role of drought-induced water-level fluctuations, especially reductions favoring cyanobacterial blooms in shallow lakes and reservoirs.

Large lakes and reservoirs in South Korea are strongly impacted by characteristic and severe monsoon rainfall events that are mainly concentrated during the summer. These water sources have a critical role in flood mitigation [35,36]. Irregularities in precipitation intensity and patterns have led to the occurrence of droughts and floods [24], which noticeably influence the flow regime, and the influx and outflux of nutrients and sediments in large lakes and reservoirs [37]. Therefore, it is imperative to understand the connections between flow regime, monsoon rainfall, nutrient regime, and general water chemistry in drinking water sources. Furthermore, it is important to explore relationships between nutrients, oxygen demanding chemicals, water temperature (WT), theoretical residence time (TRT), and the light regime, all of which are significantly impacted under drought and flood conditions.

South Korea is dependent on large reservoirs to provide drinking water to its urban populations. One such water resource is the multipurpose Andong Reservoir (AR), the second-largest artificial dam in South Korea after Soyang lake. Considering the importance of AR, we investigated the influence of monsoon precipitation, seasonal and interannual water quality fluctuations, the dynamics of dry and wet conditions, and overall long-term water quality trends. We hypothesized the spatial and seasonal water quality variability could be linked with multiple factors, including intensive rainfall events, drought, and flood periods. Further, we hypothesized that the rainfall-mediated increased inflows and outflows carry higher loads of nutrients and solids, leading to increased movement of nutrients and sediments. We probed the spatio-seasonal trends and nutrient classification criteria. Furthermore, it was assumed that nutrient enrichment status varies with the seasonal fluctuations that could help answer the critical months for regular water quality monitoring for potentially growing harmful algal species. Therefore, we also explored the associations between nutrients, Chl-a, light regime, chemical pollutants (BOD, COD), WT, and TRT. Long-term water quality trends were detected with the help of the Mann–Kendhal trend test (MKT), along with the variations in trophic status and trophic state index deviation (TSID) were also evaluated. Furthermore, we investigated the overall variations and disparities among selected water quality factors using the multivariate analysis tools of a principal component analysis (PCA) and discriminant analysis (DA).

## 2. Materials and Methods

### 2.1. Study Area

**Figure 1:**
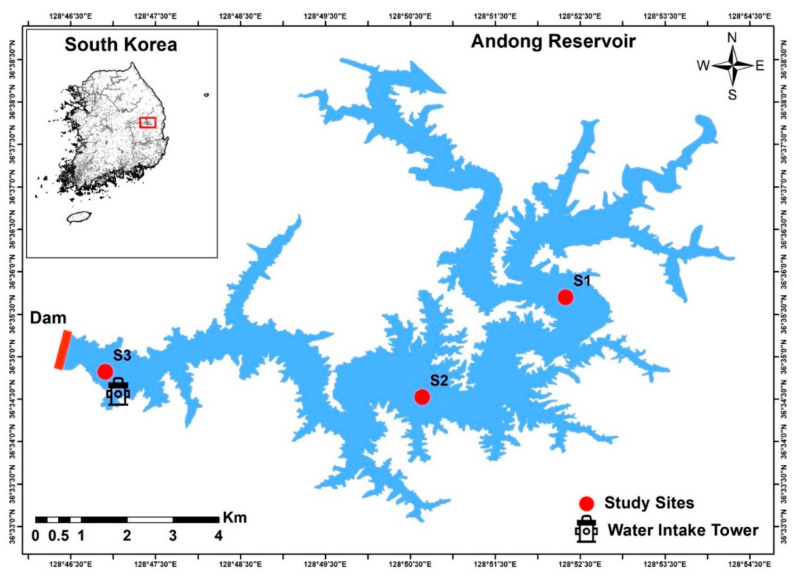
Map of study area map showing sampling sites in Andong Reservoir, South Korea.

Andong Reservoir is the second-largest multipurpose water resource in South Korea. It was constructed upstream of Nakdong River under the Korean Government’s ambitious Four Major Rivers Restoration Project (Figure 1). The AR dam has a height of 83 m and length of 612 m. The geographical, hydrological, and limnological characteristics of AR are presented in Supplementary Table S1. This long-term study was conducted for eleven years during 2010–2020 at three locations within the reservoir that were representative of its water quality. AR supply approximately 490 million m^3^ of water for domestic and industrial usage, while a further 430 million m^3^ is provided for agricultural irrigation [38,39]. This accounts for an estimated 34% of the total water use in the Nakdong River Basin, making AR the most important reservoir in South Korea. The AR basin is surrounded by mountains and forestland (81% of the basin area), while 13% is unregulated, primarily agricultural cropland [40]. Therefore, the AR plays a critical role in flood mitigation, hydroelectric power generation, recreational fishing, and water provision. Site 1 represents the reservoir part that presents the riverine features characterized more by the water flow and lower WRT. Site 2 defines the deeper water alluding to the reservoir part characterized by higher WRT. It also showed higher water clarity. Site 3 represented the water intake area located near the dam site.

### 2.2. Analyses of Water Chemistry

We obtained a water chemistry dataset from the Korean Water Environment Information System run by the Korean Ministry of Environment (MOE). The long-term water chemistry dataset ranged from January 2010 to December 2020 at three sites in the AR. We examined a total of 17 water chemistry factors, and the Secchi disk (SD) depth was also assessed. The samples were taken at three points at each site, exploring the reservoir’s surface, water column, and bottom. A complete list of all water quality parameters and their units of measurement is given in Table 1. The water samples were obtained in standard sampling bottles at a depth of 50 cm in the epilimnetic zone. The water samples were preserved in bottles that were instantly capped to limit sunlight exposure and stored in an icebox following standard procedures. The pH, dissolved oxygen (DO), WT, electrical conductivity (EC), and Chl-a level were assessed in the field using a multiprobe instrument (YSI Sonde 6600, Environmental monitoring system, Yellow Springs, OH, USA).

The TSS, COD, and BOD were estimated by the Eaton and Franson [39] method. Total nitrogen and allied chemical species (NH_4_-N, NO_3_-N) and total dissolved nitrogen (TDN) were chemically evaluated in the laboratory by the second derivative method, and then samples were digested in a persulfate solution [41,42]. Total phosphorus and associated parameters (PO_4_-P, total dissolved phosphorus (TDP)) were recorded by the ascorbic acid method, followed by the persulfate oxidation [41,43]. A total coliform bacteria (TCB) evaluation was completed following the American Public Health Association [44] method. According to the standard techniques, the nutrient-contributing parameters (TN, TP) were examined in triplicate, while BOD, COD, and TSS evaluations were performed in duplicate to ensure the reliability of the data [44,45].

### 2.3. Flood-Drought Dynamics, Flow Regime, and Rainfall Data

We collected monthly rainfall data from the local meteorological office. The average annual rainfall in South Korea is higher than the global average. The monsoon-influenced rainfall pattern results in sparse, frequent, and intense precipitation events in the reservoir watershed. We also studied the flood and drought dynamics and assessed how they could be impacting reservoir water quality. To classify any particular year as either a flood or dry year, we considered the annual rainfall intensity; if it exceeded 1400 mm, the year was a flood year, but if it was less, the year was classed as dry. The seasons were divided on the following basis: spring, March–May; summer, June–August; autumn, September–November, and winter; December–February.

### 2.4. Establishment of Trophic Status and Nutrient Enrichment

The nutrient enrichment and the trophic state of the AR were evaluated by the TSID and by studying their seasonal dynamics. The following relationships, as developed by Carlson [46] and Kratzer and Brezonik [47], were used to calculate the trophic status index (TSI) of the SD (m), TN (mg/L), TP (µg/L), and Chl-a (µg/L):

TSI (SD) = 60 − 14.41 Ln (SD)(1)

TSI (TN) = 14.43 Ln (TN) + 54.45(2)

TSI (TP) = 14.42 Ln (TP) − 4.15(3)

TSI (Chl-a) = 30.6 − 9.81 Ln (Chl-a)(4)

The TSID was determined using the associations between TSI (Chl-a) and TSI (SD), and TSI (Chl-a) with TSI (TP) in a 2D investigation. This method shows the degree of nutrient enrichment and identifies any nutrient limitations in lakes and reservoirs [48]. The nonalgal light attenuation coefficient (K_na_) was determined using the following equation [49]:

K_na_ = 1/SD − 0.025 Chl-a (5)

### 2.5. Statistical Analyses

We subjected all the datasets to the Kolmogorov–Smirnov normality test before performing log transformations. We completed the data analyses in the context of seasonal (spring, summer, autumn, and winter) and spatial variations (reservoir sites). The Mann–Kendall Test (MKT) was used to assess the prevalent trends in all water chemistry parameters that were primarily linked with human health and water usage [50]. This analysis was performed using the ProUCL version 5.1. software [51] and results were reported to be significant at a *p*-value of 0.05. A discriminant analysis (DA) was performed using SPSS software (version 22.0; SPSS Inc., Chicago, IL, USA). All graphs were constructed in SigmaPlot (ver. 14.5). A PCA/factor analysis (PCA/FA) was performed using SPSS software (version 22.0; SPSS Inc., Chicago, IL, USA). We used the PAST [52] software (Øyvind Hammer, Natural History Museum, University of Oslo, Oslo, Norway) and Sigma Plot (v. 14.5) (Systat Software Inc., San Jose, CA, USA) for all other statistical analyses.

## 3. Results and Discussion

### 3.1. Spatio-Seasonal Trends in Reservoir Water Chemistry and Nutrient Classification

The spatial and season-based physicochemical water quality evaluation provided powerful insights into the prevailing spatio-seasonal tendencies in the leading water quality parameters (Table 1). For example, the WT almost doubled from spring (7.44 ± 2.66) to summer (15.87 ± 3.19), while there was a decline from S1 to S3. There were similar WT ranges and spatial trends during spring, winter, summer, and autumn. The opposite pattern was observed for DO, which satisfied the established relationship between WT and DO. The BOD and COD spatial variations were similar during all seasons, while S1 consistently had the highest BOD and COD levels. The lowest average TP level was observed at S2 (16.79 ± 4.94) during spring, while the highest average TP loadings were observed at S1 (23.48 ± 19.8) during summer. Similarly, the average TN value was 1.58 ± 0.31 at S1 during summer, with no considerable variation.

The sestonic Chl-a spatio-seasonal variations displayed heterogeneous tendencies. The highest average level was observed at S1 (3.86 ± 0.55) during autumn, followed by S2 (2.91 ± 0.89) during summer. The lowest average Chl-a level was observed at S2 (1.57 ± 0.89) during winter. Water clarity measured as SD displayed a site-based increase from S1 to S3 during all seasons. One of our primary concerns was the bacterial populations prevailing in the reservoir water, and the TCB counts displayed significant seasonal and spatial differences. During spring and winter, the TCB loads displayed different spatial tendencies. For example, during spring, the lowest TCB average count was observed at S3 (32.21 ± 17 MPNmL^−100^), while during winter, S1 had the lowest TCB load (49.82 ± 12 MPNmL^−100^). However, the highest single-day TCB loads were recorded during summer and autumn, with values as high as 16,000 MPNmL^−100^ at S3, followed by 5400 MPNmL^−100^ at S1 during the same season.

Using reservoir nutrient loads to estimate the condition of the AR and the potential for eutrophication, we calculated the reservoir nutrient condition according to OECD [53] guidelines. The spatio-seasonal nutrient status of the AR revealed a mix of poor nutrients (PN) to average nutrients (AN) throughout the whole period of the study (Table 2). The seasonal and spatial water quality fluctuations could be influenced by multiple factors, including hydraulic WRT, nutrient loadings (internal and external), inflows and outflows, the extent of dissolved and particulate substances, and biogeochemical and photochemical processes [20,54,55]. Based on our findings, the second-largest freshwater reservoir in South Korea has an oligotrophic to mesotrophic nutrient enrichment status, and its water is suitable for consumption by humans. However, the seasonal surge in TCB may result in occasional waterborne disease outbreaks, resulting in the need to regulate the working efficiency of wastewater treatment plants (WWTPs) and mitigate the inflow of municipal sewage and industrial effluents [2,56,57].

### 3.2. Correlation Analysis of Physicochemical Water Quality

**Figure 2:**
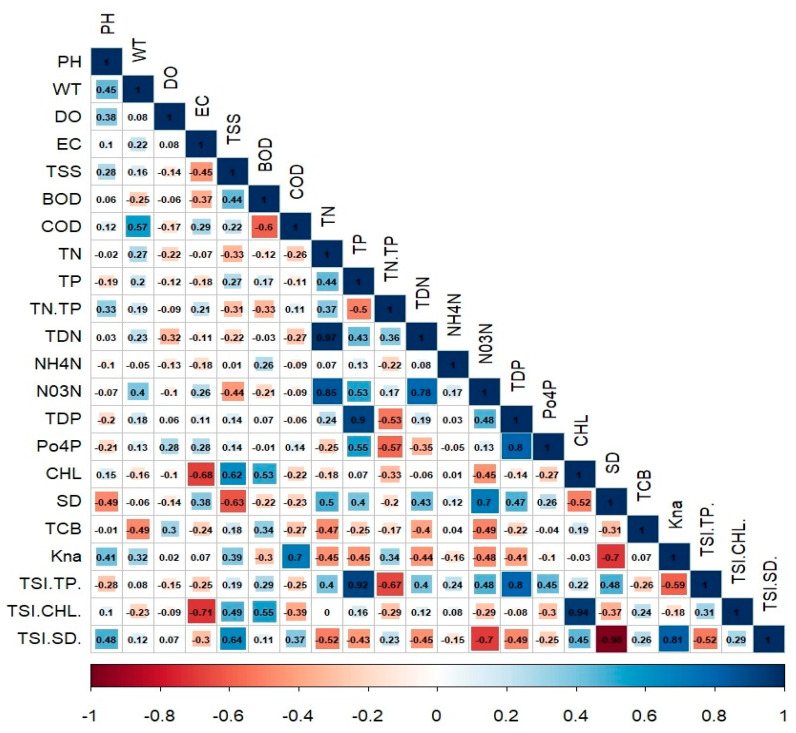
Pearson correlation analysis of water quality parameters during 2010–2020 (units mgL^−1^, except pH, WT (°C), EC (µScm^−1^), TP (µgL^−1^), Chl-a (µgL^−1^), SD (m), TCB (MPNML^−100^ and Kna (m^−1^). pH: hydrogen ion concentration; WT: water temperature; DO: dissolved oxygen; EC: electrical conductivity; TSS: total suspended solids; BOD: biological oxygen demand; COD: chemical oxygen demand; TN: total nitrogen; TP: total phosphorus; TDN: total dissolved nitrogen; NH_4_-N: ammonium-nitrogen; NO_3_-N: nitrate-nitrogen; TDP: total dissolved phosphate; PO_4_-P: phosphate; Chl: chlorophyll; TCB: total coliform bacteria; SD: Secchi depth; TCB: total coliform bacteria; K_na_: nonalgal light attenuation coefficient; TSI (TP), TSL (Chl), and TSI (SD): trophic state index of total phosphorus, chlorophyll-a, and Secchi depth, respectively.

We applied a Pearson’s correlation to evaluate the relationships and reciprocal links between all water chemistry parameters. The comprehensive correlation analysis identified various relationships among the nutrients, sestonic Chl-a, TSS, SD, TSI, and pollution indicators (Figure 2). The strength of these relationships was determined as weak (r ≥ 0.30–≤ 0.49), moderately strong (r ≥ 0.50– ≤ 0.69), or strong (r ≥ 0.7). The WT had a moderately strong (r = 0.57) association with COD, and a weak negative (r = −0.49) connection with TCB. The BOD had moderately strong positive correlations with algal Chl-a (r = 0.53) and TSI Chl-a (r = 0.55). In contrast, TSS had moderately strong positive (r = 0.62) and negative (r = −0.63) associations with algal Chl-a and SD. The nonalgal light attention coefficient (Kna) displayed a strong positive correlation with COD (r = 0.70), while it had equally negative (r = −0.45) links with TN and TP. The potential reason behind such a strong correlation with the COD levels is that the reservoir is receiving a higher inflow of organic matter, leading to higher levels of nonalgal turbidity. This could potentially lead to lower oxygen levels during higher inflows carrying loads of organic matter. The TCB had moderately strong negative links with most of the water chemistry parameters.

### 3.3. Long-Term Trends in Water Chemistry

We determined the monotonic trends of all the water quality parameters during the study period by a MKT analysis, and the results are presented in Table 3. The MKT is widely applied to evaluate increasing or decreasing tendencies and is usually reliable for long-term datasets [2,31]. This nonparametric analysis revealed that there were no trends in most of the water chemistry parameters in the AR. The WT, EC, and COD displayed an increasing trend, while there was a more severe increasing trend for COD (*S* value = 25, intercept = 2.44). In contrast, the BOD displayed a declining tendency. The Chl-a and TCB also revealed steep declines, while obligatory nutrients (TN, TP), TSS, and SD did not show any trends. It is crucial to determine the predominant water chemistry parameters for drinking water sources, as this information will indicate their sustainability as long-term water resources. The MKT can provide an understanding of the prevalent trends in critical regulatory factors. However, this nonparametric evaluation cannot extrapolate these trends into the future due to the rapidly fluctuating climate conditions resulting from global warming [58]. Our study revealed that the AR’s water quality enables its continued use as a sustainable drinking water facility. However, the tendency for a rising WT’ indicated the impact of climate change, with the potential to cause regime shifts in aquatic species living in the AR [59].

Furthermore, the tendency for the COD to increase over time pointed to the consistently rising inputs of industrial pollution to the reservoir and increases in population pressure from municipal inhabitants. The lack of a trend in most nutrients and sedimentation indicators and a declining tendency in the sestonic Chl-a indicated the availability of excellent quality drinking water [25]. It was also apparent that farmers near the AR were practicing sustainable agriculture. Overall, the MKT results provided a valuable oversight of the sustainability of the AR’s water quality.

### 3.4. Impact of Flood and Drought Dynamics

**Figure 3:**
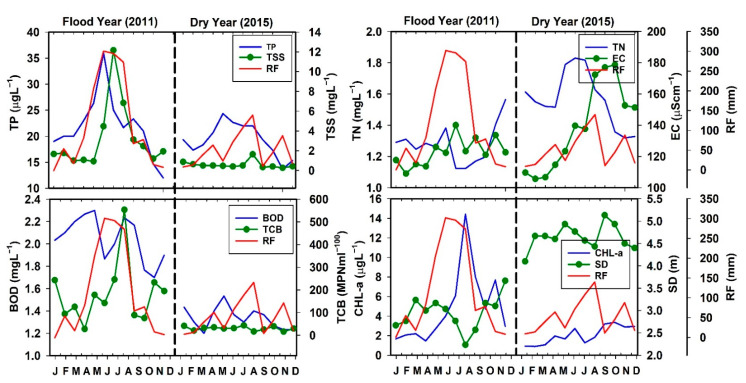
Monthly variations of TP, TSS, TN, EC, BOD, TCB, Chl-a, and SD in flood and dry years. (TP: total phosphorus, TSS: total suspended solids; TN: total nitrogen; EC: electrical conductivity; BOD: biological oxygen demand; TCB: total coliform bacteria; Chl-a: chlorophyll-a; SD: Secchi depth, and RF: rainfall).

In South Korea, the rainfall patterns vary seasonally and annually. The annual variabilities in rainfall intensity cause flood and drought conditions that could impact water quality parameters that are critical to human health and aquatic biodiversity. We observed conspicuous seasonal changes during the flood (2011) and dry (2015) years in the AR watershed, and the results are presented in Figure 3. The pattern of the total rainfall during the study period is shown in Supplementary Figure S1. The seasonal comparisons among nutrients (TN, TP), ionic regime (TSS and EC), indicators of pollution (BOD, TCB), and primary productivity (Chl-a) displayed seasonal heterogeneities during the flood and drought years. There was a close approximation between rainfall, TP, and TSS during the flood year, while the same factors displayed varying responses to the lower rainfall intensity. However, EC did not increase with the intensive rainfall events during July and August, while TN responded differently. The TN level rose during the dry year in contrast to the trends in TP and TSS. The TP in the dry year was much lower than the level recorded during the flood year. Identifying this phenomenon was sufficient to conclude that rainfall intensity had a critical influence on reservoir water quality and nutrient loadings during the flood and dry years [25,31]. It was apparent that specific RF events strongly affect water quality through the resultant delivery of high nutrient and solid loadings to the reservoir system.

We observed seasonal trends in BOD and TCB under the different precipitation intensities. These trends revealed the decisive role of rainfall as the carrier of organic pollutants, sewage, sludge, soil, human fecal matter, and garbage. Usually, high levels of BOD and TCB indicate worsening water quality, especially for human consumption; therefore, they could be used as surrogates of reservoir water quality and its suitability for human consumption. Due to the significance of BOD and TCB for human water consumption, aquatic food web health, and overall water quality, we investigated their responses to changing RF intensity during the flood and dry years. High loads could help designate the potential levels of disease-causing bacterial populations and the resulting propensity of waterborne diseases in the infant and adult human populations [24].

Like TP and TSS, the sestonic Chl-a is closely associated with the rainfall intensity during dry and flood years. As a result, there was an apparent response of water transparency with depth (i.e., SD) observed in the AR. In contrast to reports for the largest drinking water reservoir (Daecheong) in South Korea, the sestonic Chl-a exhibited a peak during the spring season of the dry year, while the BOD response was almost minimal [25,31]. Due to the rapidly changing climatic conditions, drought and flood dynamics studies are of great significance [60,61,62]. Furthermore, South Korean freshwater ecosystems are at an increased risk of damage [63]. In large lakes and reservoirs, the WRT is a critical factor that is frequently compromised under the high- and low-flows during flood and dry years, respectively. Therefore, rainfall intensity and the resultant drought and flood conditions could negatively influence the WRT, nutrient inputs, and aquatic biodiversity and cause occasional damage to waterside installations [24].

### 3.5. Relationships between Flow Regime, Nutrients, TSS, and Sestonic Chl-a

**Figure 4:**
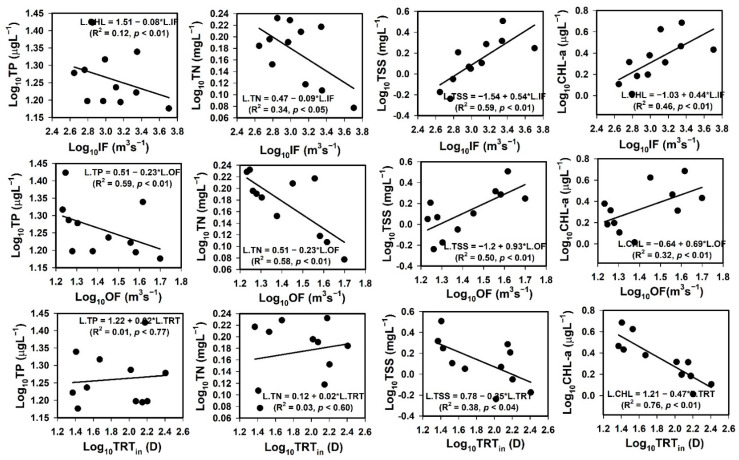
Relationships of TP, TN, TSS, and CHL-a, with inflow (IF), outflow (OF), and theoretical residence time_inflow_ (TRT_in_). TP: total phosphorus; TN: total nitrogen; TSS: total suspended solids, and Chl-a: chlorophyll-a.

We investigated the impact of flow regime (inflow, outflow, and TRT) on nutrients (TN, TP), TSS, and sestonic Chl-a, with the results indicating heterogeneous responses (Figure 4). There was a strong negative (R^2^ = 0.59, *p* < 0.01) correlation between TP and the reservoir outflow, while there was a weak negative correlation with inflow (R^2^ = 0.12, *p* < 0.01). There were moderate (R^2^ = 0.34, *p* < 0.05) to strong (R^2^ = 0.58, *p* < 0.01) negative correlations between reservoir inflow and outflow and TN, respectively. However, TRT did not substantially influence TN and TP during the study period. In contrast to the nutrient regime, TSS and sestonic Chl-a were positively correlated. There were strong positive correlations between TSS and inflow (R^2^ = 0.59, *p* < 0.01) and outflow (R^2^ = 0.50, *p* < 0.01), while Chl-a had moderately strong positive (R^2^ = 0.46, *p* < 0.01) and weak (R^2^ = 0.32, *p* < 0.01) correlations with inflow and outflow, respectively. The impact of TRT on TSS and Chl-a varied. The sestonic Chl-a was very strongly, but negatively, impacted by the TRT (R^2^ = 0.76, *p* < 0.01), while the influence of TRT on TSS was weak and negative (R^2^ = 0.38, *p* < 0.01).

We conducted a time series analysis of AR inflows and outflows under the influence of rainfall patterns, with the results indicating a strong effect of intensive monsoon rainfall events (Supplementary Figure S2). The average WRT of the river flow undergoes seasonal to diurnal changes directly associated with the transport of complex substances, physical processes, and mixing processes that regulate the riverine water within the reservoir [64]. These phenomena dictate the reservoir ecological and water quality conditions that control various biogeochemical processes [65]. The strong associations of TSS and Chl-a with inflow suggest an impending nutrient and sediment enrichment in the AR. The growing nutrient and sediment inflows and partial retention via sedimentation in large reservoirs influence downstream aquatic and terrestrial ecosystems [66,67]. Therefore, the reservoir flow regime and WRT could help develop the existing and impending links between nutrients and sedimentation. This information could also be used to create nutrient elimination procedures in large and small reservoirs [67].

### 3.6. Empirical Modelling of Nutrients and Sestonic CHL-a

**Figure 5:**
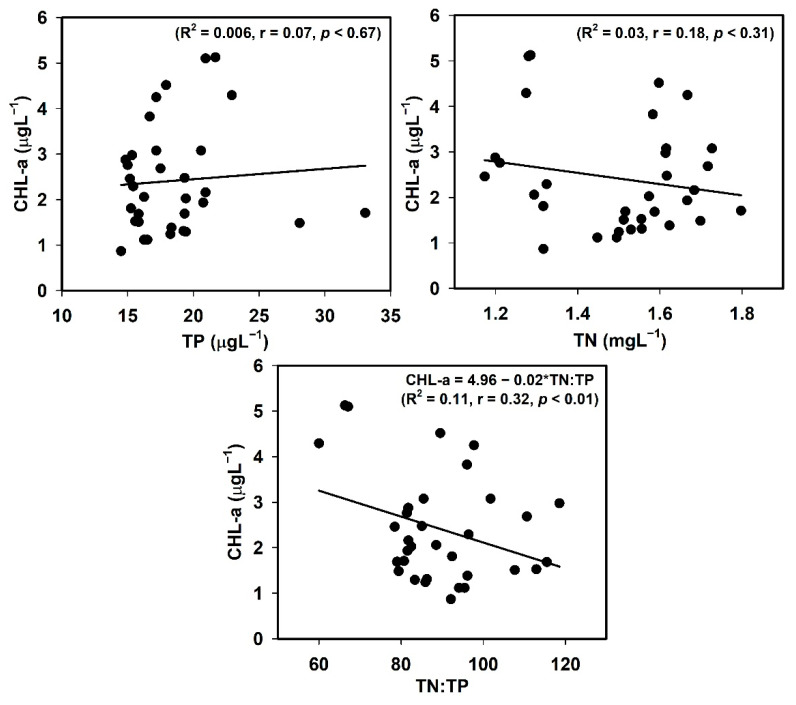
Empirical relationships between total phosphorus (TP), total nitrogen (TN), TN:TP ratio, and chlorophyll-a (Chl-a).

**Figure 6:**
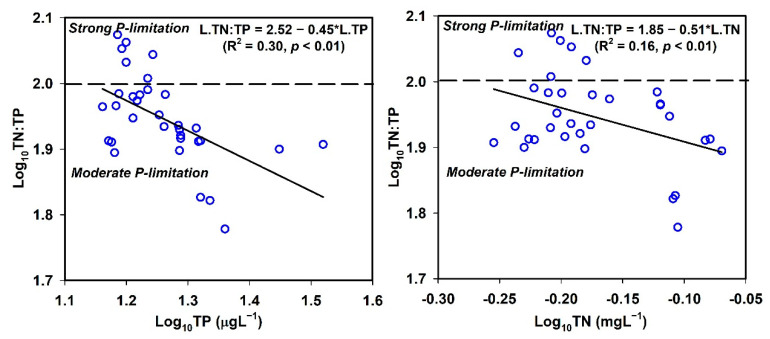
Relationships between TN:TP ratio, TP, and TN. (TP: total phosphorus; TN: total nitrogen).

To determine the most significant limiting nutrient in the AR, we evaluated the empirical links among sestonic Chl-a, nutrients, and their ambient ratios. The results indicated that the algal Chl-a could be best predicted by understanding the TN:TP ambient ratio (R^2^ = 0.11, r = 0.32, *p* < 0.01), although the relationship was too weak for certainty (Figure 5). Therefore, we further explored relationships between the N:P ratio and nutrients (TN, TP), with the results indicating a moderate P-limitation in the AR (Figure 6). The empirical relationship between the N:P ratio and TP also demonstrated the potential for a robust P-limitation scenario to develop (R^2^ = 0.30, *p* < 0.01). Therefore, P was the most limiting primary nutrient to regulate the sestonic Chl-a productivity in the AR. However, in the AR, rather than P, the most critical limiting factor was the N:P ratio, which was strongly supported by several previous studies [2,25,68,69,70]. Considering the prevalent nutrient and sestonic CHL-a trends, there was no need for immediate and strict plans to mitigate nutrient enrichment to regulate Chl-a and algal blooms in the AR.

### 3.7. Organic Pollutants, Transparency, and Nonalgal Light Attenuation

**Figure 7:**
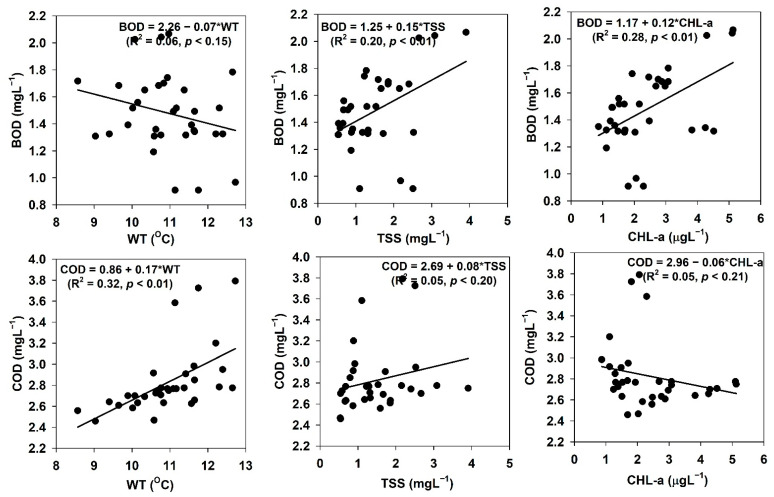
Relationships between biological oxygen demand (BOD) and chemical oxygen demand (COD) with water temperature (WT), total suspended solids (TSS), and chlorophyll-a (Chl-a) in Andong Reservoir.

**Figure 8:**
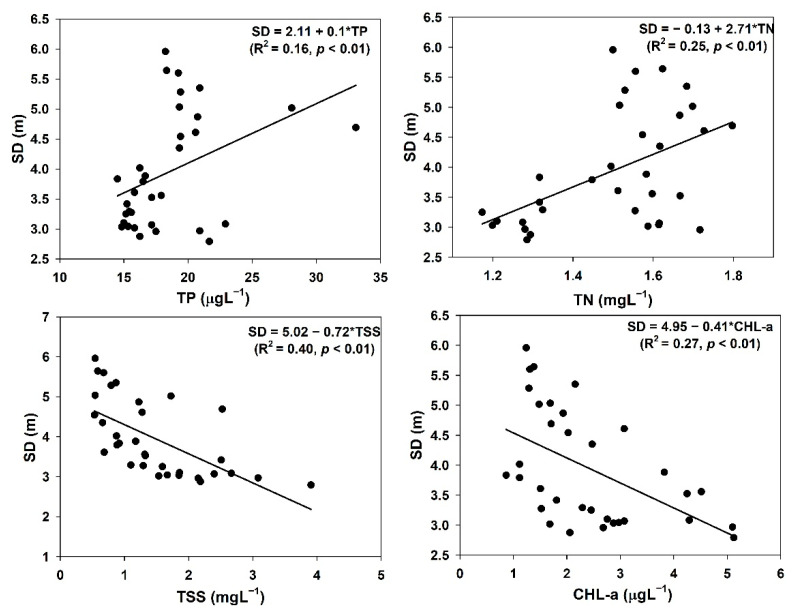
Relationships of water clarity (SD) with nutrients (TP: total phosphorus; TN: total nitrogen), total suspended solids (TSS), and algal chlorophyll (Chl-a: chlorophyll-a).

We evaluated the prevailing links between organic pollution indicators (BOD, COD), TSS, WT, and sestonic Chl-a, and the results are shown in Figure 7. The BOD showed a positive response to TSS (R^2^ = 0.20, *p* < 0.01) and a 28% linkage with the algal Chl-a (R^2^ = 0.28, *p* < 0.01). However, the COD exhibited a positive association with the WT (R^2^ = 0.32, *p* < 0.01), while it had weak negative links with algal Chl-a (R^2^ = 0.05, *p* < 0.21). The SD response to TN (R^2^ = 0.25, *p* < 0.01) was stronger than its response to TP (R^2^ = 0.16, *p* < 0.01) (Figure 8), while it had weak negative links with TSS (R^2^ = 0.40, *p* < 0.01) and algal Chl-a (R^2^ = 0.27, *p* < 0.01). To determine the critical factors affecting the SD, we further investigated the empirical links between the water quality parameters and the nonalgal light attenuation coefficient (K_na_). The results are presented in Supplementary Figure S3. Comparatively higher loads of TP (R^2^ = 0.20) than TN (R^2^ = 0.21) were confirmed in the AR due to the negative association with K_na_, as shown by the response of SD. However, the N:P ratio (R^2^ = 0.11) and TSS (R^2^ = 0.15) had a weak positive association with K_na_. There was no relationship identified between the K_na_ and sestonic Chl-a.

The BOD and COD are extensively used as indicators of organic pollutants, while SD and K_na_ highlight the subaquatic light accessibility in lentic ecosystems [31]. Light penetration is also regulated by nutrients, TSS, depth, and algal biomass [2,71,72]. The weak relationships between the light regime and other limnological factors indicated the long-term suitability of AR for drinking water supply and hydroelectric power generation. The reservoir had suitable conditions for prolonged use as a vital resource. However, further investigation is required to establish this conclusion as these findings were mainly related to BOD and COD.

### 3.8. Seasonal Multivariate Water Quality Evaluation

#### 3.8.1. The PCA/FA Results

We conducted a PCA/FA to reduce the seasonal dimensionality and identify the magnitude of variance among all water quality parameters. The dominant water quality factors influencing the reservoir water chemistry are shown in Table 4. Before conducting the PCA/FA, we completed a Bartlett test to calculate the Kaiser–Meyer–Olkin (KMO) value, which determined the suitability of the data for the PCA/FA. The Bartlett test showed that the data were appropriate, with seasonal KMO values as follows: spring, KMO = 0.58, *p* = 0.0; summer, KMO = 0.68, *p* = 0.0; autumn, KMO = 0.70, *p* = 0.0; winter, KMO = 0.65, *p* = 0.0. Furthermore, the Bartlett test had a significance value of *p* < 0.000 for all seasons, indicating some meaningful relationships among the leading water quality factors as well as revealing some of the potential factors influencing water quality in the AR watershed. We extracted two varifactors (VFs) for each seasonal PCA, with varimax rotation and fluctuating cumulative variance (CV). The CV for various seasons was calculated as follows: spring = 36.43, summer = 45.62, autumn = 42.68, and winter = 42.

The VF1 of spring accounted for 19.80% of the variance and revealed a strong positive loading (>0.70) of TP and allied chemical species, with a moderate loading of COD (0.59) and weak loading of TCB (0.47). The VF2 exhibited a strong positive TN (0.90) value and explained the impact of N-containing chemical species. During spring, there was a significant impact of agricultural activities characterized by high contributions of TN and TP to the reservoir. The VF1 of summer also indicated the dominant effect of crop farming, with strong positive loadings of nutrients (TN, TP). The VF2 (21.75% variance) had a strong positive loading of TSS (0.74), moderate positive loading of BOD (0.59), and moderate negative loading of EC (−0.54), indicating strong links between nutrients, TSS, organic pollutants, and the ionic content of inflows to the reservoir. This also verified the dominant role of intensive monsoon rainfall events during summer that mediated the transport of nutrients, solids, and organic pollutants to the AR. The VF2 also explained the moderately strong loading of sestonic Chl-a with the strong negative loading of SD (−0.68). High loadings of nutrients also characterized winter and autumn. High loadings of TN were expressed during all seasons, indicating an increasing tendency for N fertilizer use in the reservoir watershed. This may lead to high ammonia levels, which may affect the reservoir’s biota. The PCA/FA indicated the dominance of crop farming activities and increasing inflow of domestic sewage to the reservoir, which may render the AR unfit as a sustainable drinking water source.

#### 3.8.2. The DA of Seasonal Variations

A DA can categorize the dependent variables (criterion), and independent factors are used as the predictors of interval type. An accurate and appropriate estimate classification yields a high percentage (%) when a DA is applied effectively to a dataset [73]. The classification matrix produced during the DA is shown in Table 5. During this study, the four seasons represented four groups, i.e., spring, summer, autumn, and winter in the seasonal (temporal) analysis, while the three sampling stations represented the spatial analysis. We used the raw data when conducting the DA, and the discriminant function (DF) was analyzed under the standard forward stepwise and backward stepwise modes. The dependent variables included the seasons (temporal) and sites (spatial), whereas the water chemistry parameters constituted the independent variables. The standard DF mode extracted the classification matrix (CM) in 74% of cases.

**Figure 9:**
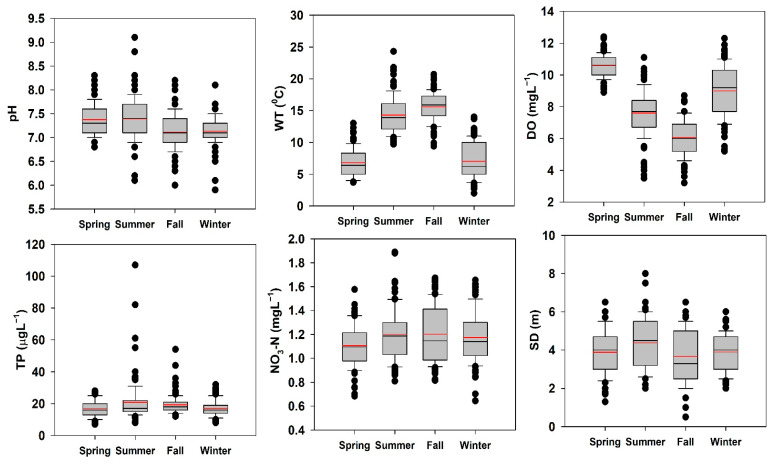
Seasonal variations of pH, WT, DO, TP, NO_3_-N, and SD in Andong Reservoir. (pH: hydrogen ion concentration; WT: water temperature; DO: dissolved oxygen; TP: total phosphorus; NO_3_-N: nitrate-nitrogen; SD: Secchi depth; spring: March–May, summer: June–August, autumn: September–November, and winter: December–February).

The seasonal DA indicated that six water chemistry parameters were significant during all four seasons in the AR (Supplementary Table S2). It also indicated that the primary source of nutrient inflow was agriculture in the AR watershed, and monsoon rainfall was the critical factor mediating the temporal and spatial changes. We displayed the seasonal trends in the chosen water quality parameters (pH, WT, DO, TP, NO_3_-N, and SD) in a box and whisker plot, as shown in Figure 9. The pH displayed a consistent decline from spring to winter, while WT displayed typical peaks during summer and autumn. The DO followed the reverse pattern. The TP loading was highest during summer, and then declined during autumn and winter. Ammonia levels were lowest during spring, while summer and autumn had similar ammonia levels. For SD, a heterogeneous response was apparent, with autumn having the lowest SD due to the higher nonalgal turbidity. High levels of TP and ammonia were encountered due to the increasing use of fertilizers in intensive agricultural practices [74]. Another potential reason for the continuously declining TP loads could be the addition of freshwater during summer [30,75].

### 3.9. Seasonal Trophic Status Assessment

**Figure 10:**
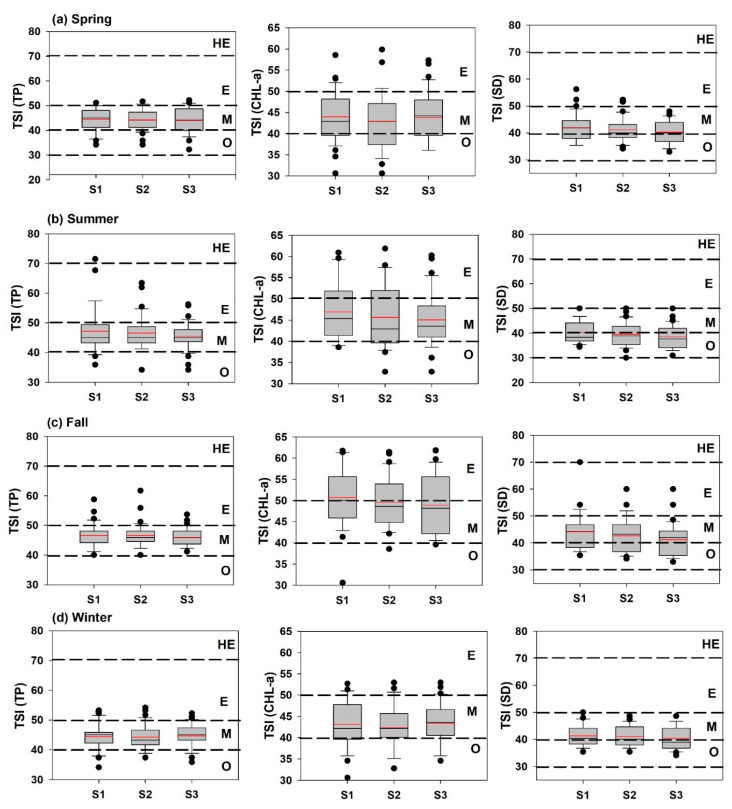
Seasonal variations of the trophic state index (TSI) of Andong Reservoir. (spring: March–May; summer: June–August; autumn: September–November, and winter: December–February; O: oligotrophic; M: mesotrophic; E: eutrophic, and HE: hypereutrophic).

Trophic state index-based evaluations of TP, CHL-a, and SD were conducted on the seasonal patterns of variation, with the results indicating a predominant mesotrophic state of nutrient enrichment (Figure 10). The TSI (TP) remained broadly similar at all sites during spring and autumn, while it declined slightly from S1 to S3 during summer, with the opposite trend during winter. In autumn, the TSI (Chl-a) indicated eutrophic conditions at S1, with a sharp fall to a mesotrophic state at sites 2 and 3. Overall, S2 had a lower trophic level for Chl-a compared to that of the other two sites. The TSI (SD) displayed a mesotrophic to oligotrophic state, with total oligotrophication during summer. There was a steady decline in the mesotrophic to the oligotrophic and near-oligotrophic state from S1 to S3.

**Figure 11:**
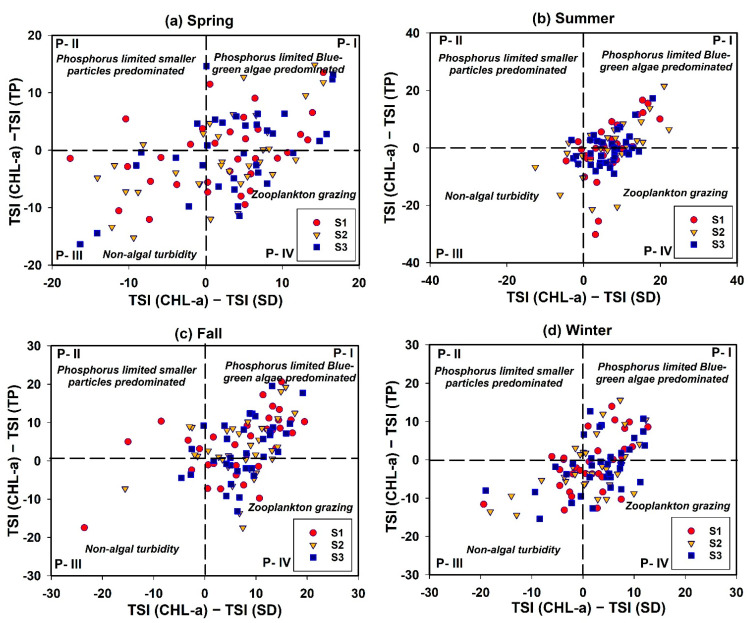
Seasonal trophic state index deviation (TSID) of Andong Reservoir (spring: March–May; summer: June–August; autumn: September–November, and winter: December–February).

From the application of the TSID, we observed spatio-seasonal heterogeneities at all sites during spring, summer, autumn, and winter, with a predominant P limitation and a tendency for zooplankton grazing (Figure 11). During spring and winter, the potential for nonalgal turbidity was higher than in that of other seasons, although the nutrient enrichment presented a scattered distribution. We observed severe zooplankton grazing, with large amounts of predominantly blue-green algae (BGA) and high P-limitation levels during summer. The pattern during autumn was similar because of the summer nutrient inflow mediated by intensive monsoon precipitation events, P-limitation, and BGA dominance.

Moderate to severe enrichment of nutrients occurs in water bodies worldwide, presenting a challenge for sustainable drinking water quality management [25,76]. The leading cause of this enhancement of eutrophication is the increasing levels of P and N in large reservoirs and natural lakes, with the primary sources of these nutrients being intensive crop production and industrial activities, mainly unregulated effluent releases from WWTPs [30,76,77]. Most the world’s large reservoirs face severe and unchecked nutrient inputs, high-turbidity, and the occurrence of harmful algal blooms [78], causing a severe water quality degradation that renders water unfit for consumption and hydroelectric power generation [24,31,79]. Although recurrent nutrient enrichment is unlikely in the RA, the possibility cannot be ignored due to large particles and the occasional predominance of BGA. Therefore, it is essential to control the high TP inflows and further investigate the phytoplankton species and their functional groups. This can be done by regulating the usage of P-yielding fertilizers and controlling industrial effluents to restrict nutrient inputs [25,80]. It is, therefore, crucial to locate the hotspots of high TP concentrations in the reservoir watershed and implement effluent standards based on individual points.

## 4. Conclusions

We conducted a multiyear (2010–2020) water quality evaluation in the second-largest multipurpose freshwater reservoir in South Korea. The Andong Reservoir (AR) is predominantly used as a drinking water resource. The study’s main aim was to assess the seasonal water quality patterns and determine whether the reservoir could be a long-term suitable drinking water resource. The outcomes mainly supported our hypothesis of the more significant influence of the rainfall-mediated flow regime impact on the leading water quality variables. The spatio-seasonal evaluation of various physicochemical water quality parameters indicated no significant organic pollutants that accompanied the sestonic Chl-a fluctuations during the different seasons. The coliform bacterial population stated the impact of municipal sewage that was likely transported through river flow.

Furthermore, the spatial and seasonal patterns of nutrients, total suspended solids (TSS), and algal Chl-a displayed heterogeneities determined by the impact of rainfall patterns. The reservoir tended to have a poor to average nutrient load throughout the study duration. The Mann–Kendhal trend test (MKT) results revealed decreasing trends in sestonic Chl-a, biological oxygen demands (BOD), and total coliform bacteria (TCB), while an increasing trend for water temperature (WT) and chemical oxygen demands (COD). The links between TSS, TP, and algal Chl-a indicated the progressive role of the monsoon in transporting nutrients and TSS to the reservoir. A comparison between dry and flood years revealed the decisive influence of the monsoon, and it also governed the reservoir flow regime. The empirical relationships indicated that TP was the most limiting factor. The N:P ratio also had a significant role and could estimate future nutrient enrichment events. Although it had the highest linkage to TN, the nonalgal light attenuation coefficient did not have a meaningful relationship with Chl-a.

The trophic status evaluation indicated the predominant mesotrophic state of nutrients, with trophic state index (TSI) (Chl-a) indicating little chance of eutrophication at S1. The Secchi disk (SD) trophic state evaluation indicated an oligo-mesotrophic state at all sites. The TSID evaluation associated the presence of large particles and blue-green algae (BGA) dominance with an indication of a moderate P-limitation, as corroborated by the empirical modeling. Furthermore, zooplankton grazing was observed in the AR during all four seasons. The multivariate analytical analysis showed that the main reason for nutrient enrichment was intensive agricultural activity in the reservoir watershed. Overall, the AR was found to have a stable and sustainable water quality status in this long-term seasonal analysis. However, being a drinking water facility, the need to control the agricultural and industrial activities and the inflow of domestic sewage through the feed river remains critical to ensure a sustainable drinking water supply. The spatial heterogeneities in WT, pH, BOD, COD, and TCB indicated that better management strategies are required to preempt the potential outbreak of waterborne diseases, especially among infants. Finally, intensive monsoon precipitation events were found to have substantially impacted the nutrient levels, TSS, and inflow of other pollutants, suggesting the need for better management of anthropic activity in the reservoir watershed.

## Figures and Tables

**Table 1:** Statistical summary of water quality parameters in Andong Reservoir. (units mgL^−1^, except pH, WT (°C), EC (µScm^−1^), TP (µgL^−1^), CHL-a (µgL^−1^), SD (m), and TCB (MPNmL^−100^). WT: water temperature EC: electrical conductivity; DO: dissolved oxygen; TSS: total suspended solids; BOD: biological oxygen demand; COD: chemical oxygen demand; TP: total phosphorus; TN: total nitrogen; Chl: chlorophyll; SD: Secchi depth; TCB: total coliform bacteria.

Water Quality Parameters	Mean ± SD (Min–Max)											
	Spring			Summer			Autumn			Winter		
	S1	S2	S3	S1	S2	S3	S1	S2	S3	S1	S2	S3
pH	7.46 ± 0.34	7.38 ± 0.33	7.29 ± 0.28	7.46 ± 0.52	7.41 ± 0.41	7.30 ± 0.45	7.20 ± 0.39	7.18 ± 0.35	6.98 ± 0.40	7.21 ± 0.30	7.18 ± 0.34	7.02 ± 0.33
	(6.8–8.2)	(6.9–8.3)	(6.8–7.9)	(6.2–9.1)	(6.6–8.8)	(6.1–8.2)	(6.5–8.2)	(6.6–8)	(6.0–7.8)	(6.1–7.7)	(5.9–8.1)	(5.9–7.7)
WT	7.44 ± 2.66	6.68 ± 2.14	6.29 ± 1.60	15.87 ± 3.19	14.45 ± 2.68	12.53 ± 1.92	16.74 ± 2.19	15.70 ± 1.91	14.34 ± 2.19	7.33 ± 3.35	6.97 ± 2.79	6.76 ± 2.63
	(3.7–13)	(4–12.3)	(3.8–9.3)	(11–24.3)	(10–21.3)	(9.7–16.8)	(12.3–20.7)	(11.6–19.0)	(9.4–17.70)	(2–14)	(3–12.2)	(2–11.1)
EC	155.9 ± 49.1	153.5 ± 47.9	155.5 ± 53.8	169.8 ± 41.1	166.5 ± 47.9	167.7 ± 53.8	157.3 ± 25.6	153.0 ± 25.7	160.7 ± 27.0	157.0 ± 50.0	156.4 ± 50.0	157.6 ± 49.5
	(103–265)	(99–255)	(96–289)	(113–287)	(119–292)	(124–305)	(122–213)	(112–204)	(123–217)	(99–324)	(99–325)	(104–306)
DO	10.73 ± 0.85	10.70 ± 0.72	10.35 ± 0.62	7.21 ± 1.70	7.69 ± 1.45	7.89 ± 1.10	6.31 ± 1.18	5.97 ± 1.15	5.90 ± 1.24	9.76 ± 1.33	8.93 ± 1.72	8.31 ± 1.49
	(9.2–12.4)	(9.5–12.3)	(8.9–11.4)	(3.7–11.1)	(3.5–10.4)	(5.5–10.2)	(3.6–8.7)	(3.2–8.7)	(4.1–8.4)	(6.9–12.3)	(5.3–11.6)	(5.2–11.2)
TSS	0.79 ± 0.42	0.88 ± 0.47	0.87 ± 0.53	2.62 ± 1.94	2.01 ± 1.87	1.23 ± 0.9	2.39 ± 1.59	2.23 ± 1.65	1.31 ± 0.87	1.14 ± 0.75	1.25 ± 1.04	1.06 ± 0.82
	(0.2–1.8)	(0.1–1.8)	(0.2–2.2)	(0.2–17.6)	(0.2–13.2)	(0.2–8.3)	(0.1–15)	(0.2–15.4)	(0.3–4.1)	(0.3–3.5)	(0.2–5.3)	(0.2–4)
BOD	1.48 ± 0.43	1.45 ± 0.42	1.46 ± 0.43	1.47 ± 0.39	1.51 ± 0.31	1.45 ± 0.36	1.63 ± 0.28	1.57 ± 0.33	1.55 ± 0.31	1.44 ± 0.36	1.42 ± 0.37	1.34 ± 0.33
	(0.6–2.3)	(0.5–2.4)	(0.5–2.3)	(0.5–2.4)	(0.9–2.1)	(0.6–2.3)	(1–2.2)	(0.4–2.1)	(0.9–2.3)	(0.8–2.2)	(0.6–2.2)	(0.7–2.1)
COD	2.87 ± 0.30	2.83 ± 0.36	2.78 ± 0.28	2.94 ± 0.51	2.91 ± 0.48	2.77 ± 0.34	3.03 ± 0.75	2.87 ± 0.52	2.73 ± 0.70	2.73 ± 0.38	2.75 ± 0.46	2.71 ± 0.34
	(2.3–3.6)	(2.1–4.1)	(2.3–3.7)	(2.3–4.6)	(2.3–4.5)	(2.3–3.6)	(2.3–5.8)	(2.2–5)	(1.9–5.7)	(2.2–3.8)	(1.7–4.5)	(2.1–4)
BOD:COD	0.52 ± 0.16	0.52 ± 0.15	0.53 ± 0.16	0.52 ± 0.16	0.53 ± 0.14	0.54 ± 0.16	0.56 ± 0.14	0.56 ± 0.14	0.60 ± 0.19	0.54 ± 0.17	0.53 ± 0.16	0.51 ± 0.15
	(0.2–0.8)	(0.1–0.8)	(0.1–0.8)	(0.1–0.8)	(0.2–0.8)	(0.2–0.8)	(0.3–0.8)	(0.1–0.8)	(0.2–1.2)	(0.2–0.9)	(0.1–0.8)	(0.2–0.8)
TP	17.09 ± 5.0	16.79 ± 4.94	16.79 ± 5.77	23.48 ± 19.8	20.70 ± 10.9	18.39 ± 6.13	19.70 ± 6.24	19.82 ± 7.56	18.36 ± 4.04	16.94 ± 5.07	16.76 ± 5.27	17.15 ± 4.43
	(8–26)	(8–27)	(7–28)	(9–107)	(8–61)	(8–37)	(12–44)	(12–54)	(13–31)	(8–30)	(10–32)	(9–28)
TN	1.46 ± 0.22	1.43 ± 0.19	1.46 ± 0.18	1.58 ± 0.31	1.50 ± 0.27	1.48 ± 0.22	1.55 ± 0.30	1.53 ± 0.26	1.50 ± 0.26	1.54 ± 0.23	1.50 ± 0.23	1.49 ± 0.20
	(1.1–2)	(1.1–1.8)	(1.2–1.8)	(1.1–2.2)	(1.1–2.1)	(1–2)	(1.1–2.4)	(1.1–2)	(1–2)	(1–2)	(1–1.9)	(1–1.9)
TN:TP	92.27 ± 28.7	91.98 ± 31	97.48 ± 35.6	86.38 ± 34.7	82.21 ± 25.7	88.52 ± 31.9	83.10 ± 19.1	82.23 ± 18.3	83.64 ± 16.6	97.91 ± 31.2	95.41 ± 26.3	92.46 ± 27.5
	(49–160)	(50–201)	(44–200)	(20–188)	(33–174)	(39–201)	(44–110)	(36–116)	(52–115)	(61–195)	(53–155)	(47–164)
Chl-a	1.88 ± 0.29	1.82 ± 0.55	1.91 ± 0.34	2.88 ± 0.84	2.91 ± 0.89	2.19 ± 0.82	3.86 ± 0.55	3.34 ± 0.24	3.28 ± 0.45	1.65 ± 0.89	1.57 ± 0.89	1.68 ± 0.84
	(0.4–6.9)	(0.3–7.9)	(0.3–6.1)	(0.9–13.8)	(0.3–21.3)	(0.5–8.2)	(0.4–9.6)	(0.9–9.3)	(1–9.7)	(0.4–3.8)	(0.2–3.9)	(0.3–3.9)
SD	3.69 ± 1.17	3.85 ± 1.09	4.09 ± 1.18	4.08 ± 1.15	4.36 ± 1.42	4.65 ± 1.43	3.35 ± 1.30	3.65 ± 1.42	3.99 ± 1.51	3.78 ± 0.94	3.84 ± 0.96	4.06 ± 1.15
	(1.3–5.5)	(1.7–6)	(2.3–6.5)	(2–6)	(2–8)	(2–7.5)	(0.5–5.5)	(1–6)	(1–6.5)	(2–5.5)	(2.2–5.5)	(2.2–6)
TCB	49.76 ± 12	43.64 ± 14	32.21 ± 17	200.36 ± 25	229.52 ± 51	148.67 ± 46	276.33 ± 88	266.09 ± 33	765.00 ± 55	49.82 ± 12	79.12 ± 14	72.64 ± 16
	(0–345)	(0–180)	(0–136)	(1–1600)	(7–1600)	(5–920)	(2–5400)	(0–4300)	(1–16000)	(0–300)	(1–920)	(3–920)

**Table 2:** Nutrient classification of Andong Reservoir according to OECD [53].

**Condition of Reservoir**	**Chl-a (µgL^−1^)**	**Seasons**	**Chl-a (µgL^−1^)**		
			**Sites**		
Lack of nutrients (LN)	<1		S1	S2	S3
Poor nutrients (PN)	<2.5	Spring	1.88 (PN)	1.82 (PN)	1.91 (PN)
Average nutrients (AN)	2.5–8.0	Summer	2.88 (AN)	2.91 (AN)	2.19 (PN)
Eutrophication (E)	8.0–25.0	Autumn	3.86 (AN)	3.34 (AN)	3.28 (AN)
Super eutrophication (SE)	>25	Winter	1.65 (PN)	1.57 (PN)	1.68 (PN)

**Table 3:** Long-term trend analysis of water quality parameters in the Andong Reservoir during 2010–2020 (units mgL^−1^, except pH, WT (°C), EC (µScm^−1^), TP (µgL^−1^), Chl-a (µgL^−1^), SD (m), TCB (MPNML^−100^). pH: hydrogen ion concentration; WT: water temperature; EC: electrical conductivity; DO: dissolved oxygen; TSS: total suspended solids; BOD: biological oxygen demand; COD: chemical oxygen demand; TP: total phosphorus; TN: total nitrogen; TDP: total dissolved phosphate; PO_4_-P: phosphate; NO_3_-N: nitrate-nitrogen; NH_4_-N: ammonium-nitrogen; TDN: total dissolved nitrogen; Chl: chlorophyll; SD: Secchi depth; TCB: total coliform bacteria).

Water Quality Parameters	*S* Value	*p* Value	Slope	Intercept	Trend
pH	−5	0.38	−0.001	7.24	No trend
WT	36	0.00	0.15	10.02	Increasing
EC	31	0.00	8.38	108.95	Increasing
DO	−1	0.50	0.0002	8.31	No trend
TSS	−17	0.10	−0.10	2.13	No trend
BOD	−31	0.00	−0.06	1.88	Decreasing
COD	25	0.03	0.06	2.44	Increasing
TP	−10	0.24	−0.13	19.29	No trend
TN	−3	0.43	0.006	1.46	No trend
TN:TP	17	0.10	1.51	80.38	No trend
TDP	3	0.44	0.0001	0.01	No trend
PO_4_-P	5	0.38	0.0003	0.006	No trend
NO_3_-N	15	0.13	0.01	1.06	No trend
NH_4_-N	−15	0.13	−0.001	0.02	No trend
TDN	−9	0.26	0.0001	1.36	No trend
Chl-a	−35	0.00	−0.26	4.0	Decreasing
SD	17	0.11	0.08	3.45	No trend
TCB	−39	0.00	−66.70	584.63	Decreasing

**Table 4:** Principal component analysis (PCA) of water quality parameters (units mgL^−1^, except pH, WT (°C), EC (µScm^−1^), TP (µgL^−1^), Chl-a (µgL^−1^), SD (m), and TCB (MPNML^−100^); varimax rotation method, bold and italic text represents strong (>0.70) and moderate positive loadings (0.5–0.70), respectively. pH: hydrogen ion concentration; WT: water temperature; DO: dissolved oxygen; EC: electrical conductivity; TSS: total suspended solids; BOD: biological oxygen demand; COD: chemical oxygen demand; TN: total nitrogen; TP: total phosphorus; TDN: total dissolved nitrogen; NH_4_-N: ammonium-nitrogen; NO_3_-N: nitrate-nitrogen; TDP: total dissolved phosphate; PO_4_-P: phosphate; CHL-a: Chlorophyll-a; TCB: total coliform bacteria; SD: Secchi depth. (Spring: KMO = 0.58, *p* = 0.0; Summer: KMO = 0.68, *p* = 0.0; Autumn: KMO = 0.70, *p* = 0.0; Winter: KMO = 0.65, *p* = 0.0).

Water Quality Factors	Spring		Summer		Autumn		Winter	
	VF1	VF2	VF1	VF2	VF1	VF2	VF1	VF2
pH	−0.05	−0.25	−0.33	−0.02	−0.22	0.03	−0.17	0.01
WT	−0.17	−0.08	0.21	0.21	0.04	0.27	−0.12	0.44
DO	0.24	−0.06	−0.10	−0.14	−0.33	−0.26	0.19	−0.43
EC	−0.19	−0.47	−0.12	*−0.54*	0.26	−0.02	**0.71**	0.14
TSS	0.01	0.23	0.32	**0.74**	−0.32	0.21	*−0.52*	0.07
BOD	*0.59*	0.25	−0.07	*0.61*	−0.19	0.05	−0.31	0.15
COD	−0.07	−0.26	0.26	0.00	−0.23	*0.53*	0.16	−0.07
TN	−0.19	**0.90**	**0.81**	−0.22	**0.92**	0.11	0.19	**0.93**
TP	**0.82**	0.41	**0.80**	0.47	0.32	**0.88**	**0.83**	0.12
TN:TP	**−0.89**	−0.06	−0.28	−0.71	0.36	**−0.84**	*−0.68*	0.40
TDN	−0.17	**0.92**	**0.78**	−0.20	0.90	0.05	0.12	**0.94**
NH_4_-N	0.03	0.24	−0.02	*0.61*	−0.15	0.12	0.21	−0.41
NO_3_-N	0.11	0.52	**0.86**	−0.23	**0.91**	0.16	0.49	**0.73**
TDP	**0.84**	0.17	**0.79**	0.44	0.18	**0.91**	**0.90**	0.06
PO_4_-P	**0.75**	−0.32	*0.65*	0.43	−0.12	**0.81**	**0.76**	−0.18
Chl-a	0.00	0.43	−0.18	*0.62*	*−0.56*	0.00	−0.43	0.06
SD	0.23	0.13	0.26	*−0.68*	**0.74**	−0.13	*0.60*	0.27
TCB	0.47	−0.20	−0.29	0.43	−0.33	−0.19	−0.11	−0.08
Eigenvalues	3.56	2.99	4.30	3.92	4.16	3.52	4.38	3.18
% of variance	19.80	16.64	23.87	21.75	23.10	19.57	24.34	17.65
Cumulative %	19.80	36.43	23.87	45.62	23.10	42.68	24.34	42.00

**Table 5:** Classification matrix for discriminant analysis (DA) of temporal variations in Andong Reservoir water quality (spring: March–May; summer: June–August; autumn: September–November; and winter: December–February).

	% Correct		Season Assigned by the DA		
Stepwise Mode		Spring	Summer	Autumn	Winter
Spring	81.8	81	2	0	23
Summer	72.7	4	72	18	7
Autumn	77.8	0	25	77	6
Winter	63.6	14	0	4	63
Total	74	99	99	99	99

## Data Availability

The data maybe available upon request to the corresponding author, however, it is with subject to approval from the funding agency.

## Supplementary Materials

The following are available online at https://www.mdpi.com/article/10.3390/ijerph182010714/s1, Table S1: The geographic, hydrological, and limnological features of Andong Dam. Figure S1: Total rainfall pattern of Andong Reservoir. Figure S2: Relationship of inflow, outflow and rainfall in Andong Reservoir from 2010–2020. Figure S3: Influence of TP (total phosphorus), TN (total nitrogen), TN:TP ratios, TSS (total suspended solids) and CHL-a (chlorophyll-a) on non-algal light attenuation coefficient (Kna). Table S2: Supplementary Table S2. Classification functions for discriminant analysis of seasonal variations in water quality of the Andong Reservoir.
