# Supplementary material for: Seasonal Water Quality and Algal Responses to Monsoon-Mediated Nutrient Enrichment, Flow Regime, Drought, and Flood in a Drinking Water Reservoir
Source: Int J Environ Res Public Health. 2021 Oct 13;18(20):10714. doi: 10.3390/ijerph182010714 (PMC8535919; doi:10.3390/ijerph182010714)
Supplement: Supplementary file 1 [file ijerph-18-10714-s001.zip › ijerph-1395891-supplementary.pdf]

<u>**Supplementary Material**</u>

Supplementary Table S1. The geographic, hydrological, and limnological features of Andong Dam.

**Attributes**	**Characteristics**
Construction period (years)	1971-1976
Dam type	Embankment and rock-fill
Height of dam (m)	83
Length of dam (m)	612
Mean depth (m)	19.4
Maximum depth (m)	60
Surface area (km^2)	60
Total drainage area (km^2)	1584
Average annual rainfall (mm)	950
Total capacity (m^3)	1248×10^6
Active capacity (m^3)	10×10^8
Hydroelectric power generation (MW)	90

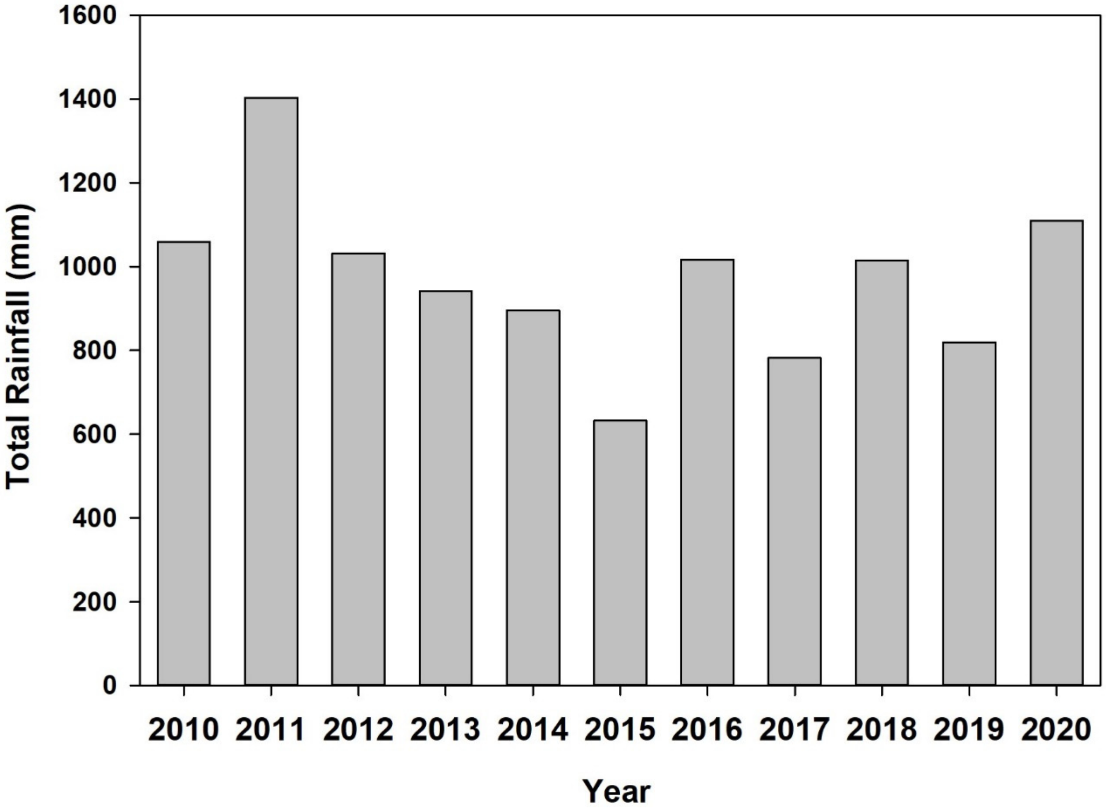

Supplementary Figure S1. Total rainfall pattern of Andong Reservoir.

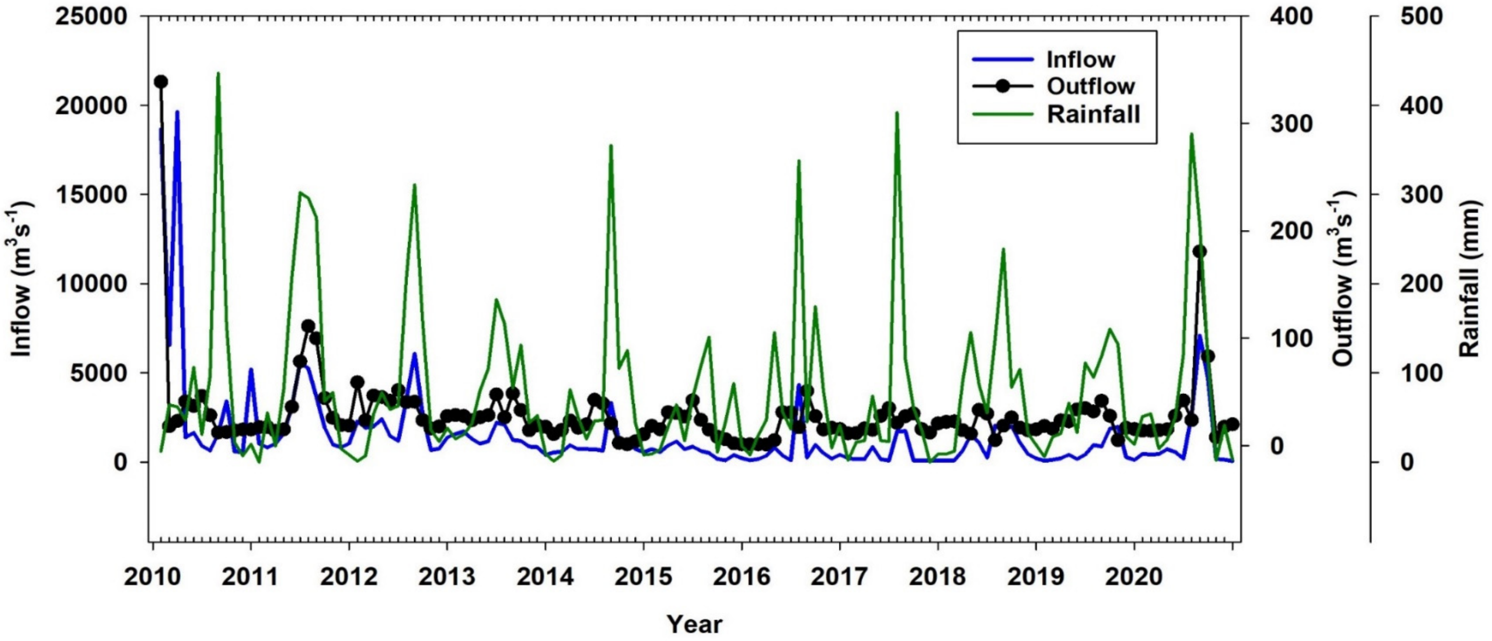

Supplementary Figure S2. Relationship of inflow, outflow and rainfall in Andong Reservoir from 2010-2020.

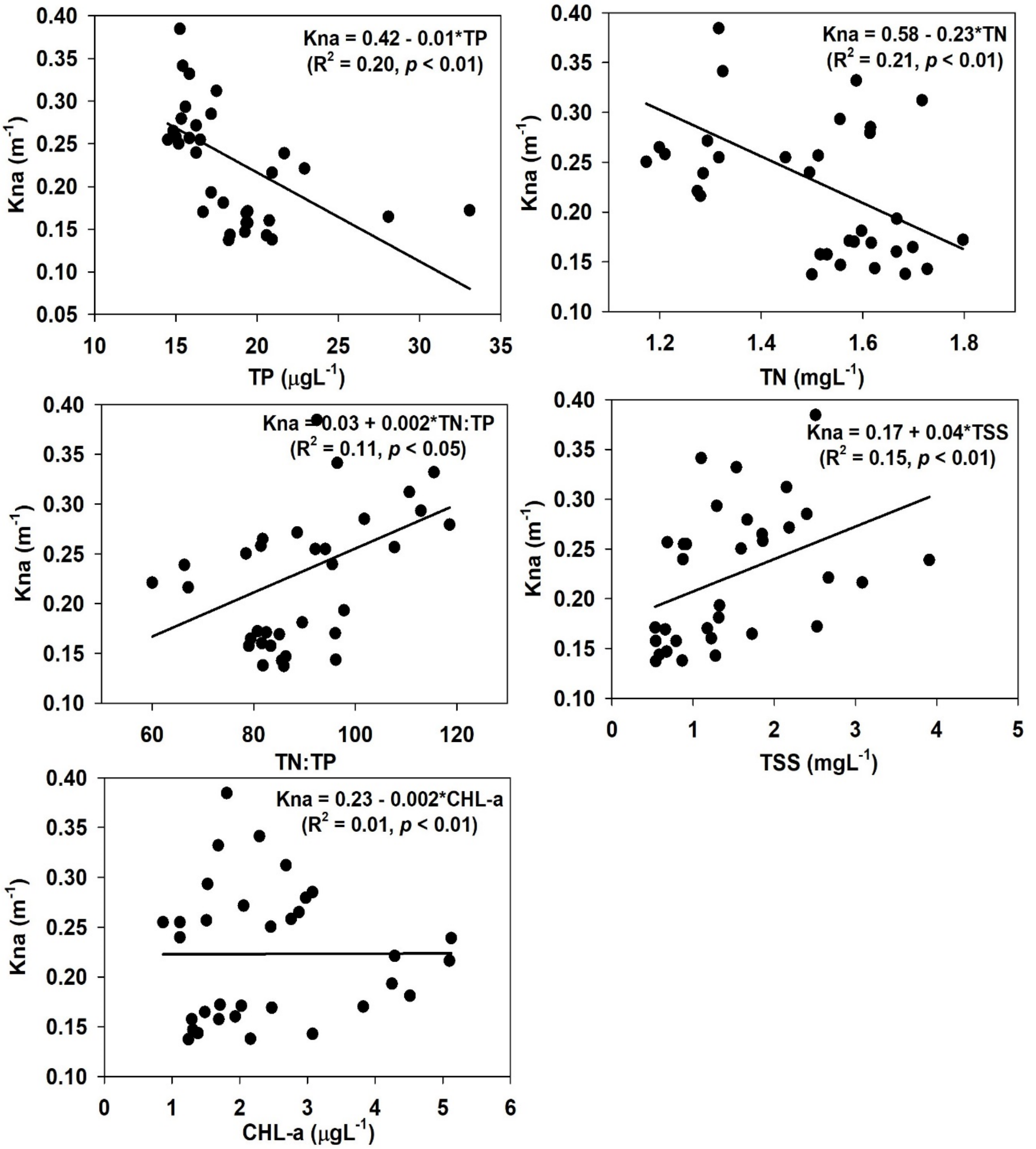

Supplementary Figure S3. Influence of TP (total phosphorus), TN (total nitrogen), TN:TP ratios, TSS (total suspended solids) and CHL-a (chlorophyll-a) on non-algal light attenuation coefficient (Kna).

Supplementary Table S2. Classification functions for discriminant analysis of seasonal variations in water quality of the Andong Reservoir. pH: hydrogen ion concentration, WT: water temperature, DO: dissolved oxygen, EC: electrical conductivity, TSS: total suspended solids, BOD: biological oxygen demand, COD: chemical oxygen demand, TN: total nitrogen, TP: total phosphorus, TDN: total dissolved nitrogen, NH_4-N: ammonium-nitrogen, NO_3-N: nitrate-nitrogen, TDP: total dissolved phosphate, PO_4-P: phosphate, CHL-a: Chlorophyll-a, TCB: total coliform bacteria, SD: Secchi depth, Spring: March-May: Summer: June-August, Fall: September-November, and Winter: December-February) (Fisher's linear discriminant functions).

Used variables	Spring	Summer	Fall	Winter
pH	52.75	53.24	51.15	51.49
WT	2.60	3.60	3.49	2.27
DO	7.86	7.07	6.11	6.62
EC				
TSS				
BOD				
COD				
TN				
TP	0.46	0.52	0.46	0.42
TN:TP				
TDN				
NH_4-N				
NO_3-N	12.99	9.09	11.39	15.93
TDP				
PO_4-P				
CHL-a				
SD	5.53	6.53	5.76	5.17
TCB				
(Constant)	-268.23	-275.85	-250.92	-245.87
